# Seasonal Variations of Polyphenols Content, Sun Protection Factor and Antioxidant Activity of Two Lamiaceae Species

**DOI:** 10.3390/pharmaceutics13010110

**Published:** 2021-01-16

**Authors:** Juliana de Medeiros Gomes, Márcio Vinícius Cahino Terto, Sócrates Golzio do Santos, Marcelo Sobral da Silva, Josean Fechine Tavares

**Affiliations:** Laboratório Multiusuário de Caracterização e Análise, Universidade Federal da Paraíba, João Pessoa, PB 58051-900, Brazil; juliana@ltf.ufpb.br (J.d.M.G.); cahino@ltf.ufpb.br (M.V.C.T.); socratesgolzio@ltf.ufpb.br (S.G.d.S.); marcelosobral.ufpb@gmail.com (M.S.d.S.)

**Keywords:** photoprotection, *M. villosa*, *P. amboinicus*, ultraviolet radiation, PCA, seasonality

## Abstract

Secondary metabolite production by plants is influenced by external environmental factors that can change depending on the seasons, which makes it important to know how the plant, through its metabolism, is able to adapt to these variations*. Mentha x villosa* and *Plectranthus amboinicus* present in their chemical composition polyphenols, and through previous studies, it has been seen that these two species present promising in vitro photoprotective activity. The aim of this study was to evaluate seasonal alterations in photoprotective and antioxidant activities and the influence of factors such as precipitation levels and sun radiation incidence. Thus, polyphenol quantification, cromatographics (HPLC-DAD) and multivariate (PCA) analyses of extracts of the two species through twelve months were done. It was observed that the best months for photoprotective and antioxidant activities were September for *M. villosa* and July for *P. amboinicus* (SPF = 14.79). It was possible to conclude that solar radiation more clearly influences the production of phenolics and the increase of SPF in *M. villosa,* in addition to favoring the antioxidant activity of the two species, while precipitation seems to have no influence.

## 1. Introduction

Seasonal variations of secondary metabolites produced by plants, both qualitatively and quantitatively, can occur throughout the year in response to modifications in plant environmental conditions, including climatic [1,2]. This variation can also modify the biological activity that plant extracts have, since samples of the same plant collected in different months will present, or not, some compounds, as well as their concentration can also be different, thus, the extract can even present different pharmacological properties throughout the year [3,4].

It is known that an increase of UVB radiation incidence and a decrease of precipitation can cause an increase in the production of free radicals, causing cellular damage that forces the plant to respond to this stimulus, altering morphological characteristics until the content of metabolites produced change, i.e., the increase of polyphenols [5]. Therefore, by evaluating environmental factors such as temperature, water availability, solar radiation incidence, among others, we can better understand the metabolic alterations happening in plants [6,7,8].

*Mentha x villosa* Hudson and *Plectranthus amboinicus* (Lour.) Spreng are two species from the Lamiaceae Family that, in Brazil, that are popularly called “hortelã da folha miúda” and “hortelã da folha grossa”, respectively. They are used in folk medicine mainly as antimicrobials, but *M. x villosa* is also used in the form of infusion or decoction of its leaves to treat stomach problems and menstrual cramps, in addition to being used as a sedative [9] and an antiparasitic [10]. Scientifically, its endothelium-dependent hypotensive and vasorelaxant effects have been demonstrated [11], as well as its antimicrobial, antinociceptive [12], antitumor [13], anti-inflammatory [14] and antioxidant activities [15,16]. *P. amboinicus* is used for the treatment of colds, coughs, asthma, and diseases of the respiratory tract in general, as well as to treat headaches, fever, skin diseases, and gastrointestinal disorders [17,18,19]. Thus, because it is a well-known species and used by the population, many studies on its effects already exist and demonstrate its antimicrobial and antiviral activities in vitro [20,21], activity against respiratory and gastrointestinal disorders, anticonvulsant, antitumor [22,23], analgesic, anti-inflammatory, and antioxidant activities [24,25]. These two species are also used in culinary as flavorings and in the preparation of some foods [17,26].

Many of the biological activities presented by *M. villosa* and *P. amboinicus* are due to the presence of essential oils that are widely studied and have monoterpenes and sesquiterpenes in their composition [17,27]. However, its nonvolatile extracts also have a large amount of biologically interesting compounds, such as phenolic compounds. In *M. villosa*, several phenolic acids have been identified, such as rosmarinic acid, quinic acid, and chlorogenic acid, as well as several flavonoids, such as luteolin and apigenin derivatives, hesperidin, kaempferol-3-O-glucuronide, eriocitrin and chrysoeriol-7-O-rutinoside [28]. In *P. amboinicus*, many polyphenols have also been identified, such as caffeic acid, gallic acid, rosmarinic acid, crisimaritin, luteolin, and apigenin derivatives, p-cumaric acid, taxolifin, among others [29].

Therefore, these species present in their chemical composition an interesting quantity of phenolic compounds, that are a secondary metabolite class mainly known because of their anti-inflammatory, antioxidant and photoprotective properties [30,31,32]. Their antioxidant properties are closely linked to their stable chemical structures, making them capable of neutralizing reactive oxygen species (ROS), inhibiting lipid peroxidation, and even preventing the production of free radicals [33].

A relevant property attributed to this metabolite class is the solar protection activity, since they are capable of filtering the incident ultraviolet radiation. Therefore, when UVB and UVA rays intensity is high, plants can be stimulated to produce phenolic compounds so they can absorb or disperse solar energy and make it harder to damage plant tissues [34,35].

*M. villosa* and *P. amboinicus* have rosmarinic acid (RA) as the majority compound in their ethanolic extracts. Studies related to photoprotective activity of this acid have showed a favorable future for this metabolite, since its photoprotective capacity has been observed, besides having good antioxidant activity and contributing to lipid peroxidation inhibition [36,37,38], which places it in a relevant position in the development of new sunscreens.

Thus, based on the extensive literature demonstrating *M. x villosa* and *P. amboinicus* therapeutic aspects, these two species deserve attention, mainly in areas where they are not well explored, such as in photoprotection and seasonality of nonvolatile extracts, since plants employ many mechanisms to adapt themselves to its environmental conditions in order to regulate its metabolism [39] and it can directly impact in secondary metabolites production. Therefore, understanding the annual variations of secondary metabolites of these two species can help us to better use them in terms of pharmaceutical product development. A previous study by Terto et al. [40], as well as unpublished data [41], showed that these two species have promising photoprotective activity in vitro since they presented a sun protection factor (SPF) of around 13. Thus, we evaluated the seasonal variations that occurred in *M. x villosa* and *P. amboinicus*, by monitoring the quantitative production of polyphenols, flavonoids, and rosmarinic acid month by month, the role of solar radiation, and precipitation in the production of these metabolites and their implications for the SPF and antioxidant activity of these extracts.

## 2. Materials and Methods

### 2.1. Plant Material

Aerial parts of *Mentha x villosa* Hudson and *Plectranthus amboinicus* (Lour.) Spreng., Lamiaceae, were collected at 8 am on the 20th of every month, from January to December 2019, where they were cultivated at the Federal University of Paraiba, Institute of Pharmaceuticals Research (coordinates 7°8′29.875″ S/34°50′48.757″ W), Campus I, João Pessoa, PB. A specimen of *M. villosa* was placed in the Herbário Prisco Bezerra, from the Federal University of Ceará, Fortaleza—CE, under n 14.996. This plant is registered at the SISGEN (National System of Genetic Heritage Management and Associated Traditional Knowledge) platform under reference number A3BA60D. A specimen of *P. amboinicus* species is deposited at Lauro Pires Xavier Herbarium (JPB/UFPB) under number identification JPB0047239 and its register at SISGEN is under reference number AAB0FA6. 

### 2.2. Preparation of Crude Ethanol Extracts

Fresh aerial parts (500 g) of *M. villosa* and *P. amboinicus* were crushed and submitted to maceration in 96% ethanol for three consecutive days; this process was repeated three times. 4 l of ethanol was used in the maceration process of *M. villosa* and 3 l of ethanol for *P. amboinicus*. After maceration, extracted solutions were concentrated using rotary evaporator equipment at 40 °C to obtain the crude ethanolic extracts (CEE) of the two species, weighing approximately 11.5 g for *M. villosa* and 12.37 g for *P. amboinicus*. The maceration process was repeated every month of 2019 right after aerial parts harvest, resulting in 12 *M. x villosa* ethanolic extracts and 12 *P. amboinicus* ethanolic extratcs. All of these extracts were used in every test made.

### 2.3. Reagents and Equipment

Solvents used were HPLC-grade methanol (Tedia^®^, Rio de Janeiro, Brazil), formic acid (J. T. Baker^®^, Aparecida de Goiânia, Brazil), acetic acid (J. T. Baker^®^, Aparecida de Goiânia, Brazil), phosphoric acid (Proquimios^®^, Rio de Janeiro, Brazil), and type I water obtained by a purification system (Milli-Q-Millipore^®^), besides absolute ethanol (Neon^®^, Suzano, Brazil), Polawax^®^ cream (João Pessoa, Brazil), Folin-Ciocalteu reagent, 1-1-diphenyl-2-picrylhydrazyl (DPPH) (Sigma-Aldrich, São Paulo, Brazil), aluminum chloride (AlCl_3_) and Rosmarinic acid (RA) obtained from Sigma Aldrich^®^, São Paulo, Brazil, with 96% of purity.

The used equipments were a UV-visible spectrophotometer (UV-2550, Shimadzu^®^, Barueri, Brazil) and an HPLC system from Shimadzu^®^ (prominence) equipped with LC-20AT quaternary solvent pumping module, SIL-20A HT auto-injector, DGU-20A5R degassing system, CTO-20A column oven, detector SPD-M20A diode array and CBM-20A controller. The column used was Kromasil^®^ C18 (250 mm × 4.6 mm a.i. filled with 5 μm particles) (Sigma Aldrich^®^, São Paulo, Brazil), with SecurityGuard Gemini^®^ C18 pre-column (4 mm × 3.0 mm a.i. filled with 5 μm particles). The LC Solution^®^ software (Shimadzu^®^, Barueri, Brazil) was used for equipment control, data acquisition, and analysis.

### 2.4. HPLC Analytical Chromatographic Methods

The method used for quantification of RA in *M. villosa* begins at 38% of solvent B, reaching 42% in 5 min and remains at this concentration until 9 min. From 9 to 12 min, the gradient is altered from 42 to 45% of solvent B and it reaches 50% in 15 min. From 15 to 17 min, the gradient returns to 45%, and it remains unchanged until 20 min, when it returns to the initial condition at 38% of mobile phase B, and the run stops at 24 min at this concentration. The flow rate used was 1 mL/min, oven temperature at 26 °C, the injection volume of 20 µL, the detection was performed at 330 nm by a diode array UV (DAD). In addition, extract and standard samples were made in triplicate and filtered with the diluent solution at a concentration of 50% : 50% MeOH : acidified water (0.1% of H_3_PO_4_).

For extract samples of *P. amboinicus*, a diluent solution used was MeOH:acidified water (0.1% of formic acid) 1:1, the run starts at 50% of solvent B and reaches 60% in 20 min, returning to 50% in 21 min and remaining at this concentration until the end, at 26 min. In this case, the flow rate used was 0.6 mL/min [40].

### 2.5. Solar Radiation and Precipitation Treatments

The exposure consisted of submitting plants to naturally occurring solar radiation and precipitation during 2019 at their harvest place at the Institute of Pharmaceuticals Research—UFPB and collecting adult plants to observe changes in the content of investigated compounds among each month of harvest. Solar radiation incidence and precipitation data were collected on the website (https://portal.inmet.gov.br/) of the National Meteorologic Institute of Brazil (INMET).

### 2.6. Total Polyphenol Content Determination

In this assay [42], 120 μL of samples (from the two plants, for each month) at a concentration of 1 mg/mL were treated with 500 μL of Folin–Ciocalteu (10%) reagent and gallic acid was used as standard. Time reaction was 8 min, and at this moment, the reaction was maintained at rest. Later, 400 μL of sodium carbonate (7.5%) was added to neutralize the mixture. Then, triplicate samples were kept at room temperature, in the dark for 120 min. Meanwhile, they were transferred to 96 well plates for later reading on a UV-visible spectrophotometer (UV-2550, Shimadzu) at 765 nm.

Linear regression was used to calculate the total phenolic content of samples, it was made through gallic acid calibration curve (25, 50, 75, 100, 150 and 200 μg/mL), and results were expressed as mg GAE/g of the sample.

### 2.7. Rosmarinic Acid Quantification

RA quantification was made through the construction of a calibration curve using the standard concentrations of 5.6, 11.25, 22.5, 45 and 90 µg/mL for extracts of both species. Samples of each month were prepared in triplicate, at a concentration of 1 mg/mL and were injected in HPLC according adequate analytical method described.

### 2.8. Flavonoids Total Content Determination

Flavonoids content was evaluated by the spectrophotometric method proposed by Schmidt and Ortega, [43] with adaptations, using aluminum chloride (AlCl_3_) as a reagent. Thus, 0.1 mL of AlCl_3_ (2.5%) was added to 0.1 mL of samples (1 mg/mL) in 96 well plates. The mixture was kept away from light for 30 min, and later, absorbance was measured at 410 nm, using a spectrophotometer UV-Visivel (UV-2550, Shimadzu) [44]. The assay was made in triplicate and flavonoids total content was calculated through a linear regression equation obtained from the quercetin calibration curve (5; 25; 50; 100 and 200 μg/mL). Results were expressed as mg of quercetin/g of sample.

### 2.9. Determination of Antioxidant Activity

DPPH method was applied for this assay [45], using methanol as a solvent. Thus, in 96 well plates, DPPH solution at 0.3 mM (100 μL) was added to 100 μL of different concentrations of extracts of both plants studied (usually 10, 20, 40, 80, and 160 μg/mL were used, but in certain samples, it was also necessary to use 320 μg/mL). This reaction remained at rest and away from light for 30 min and subsequently, the reading was done on a spectrophotometer UV-Visivel (UV-2550, Shimadzu) at 518 nm. This assay was performed on samples of all months in triplicate and, free radical scavenging activity (SA) of each concentration used was calculated by the following Equation (1). After calculating SA, calibration curves were done and results were expressed as CE_50_.


SA (%) = (A_negative control_ − A_sample_)/A_negative control_ × 100 (1)

Equation (1) Free radical scavenging activity formula.

where,

SA (%) = percentage of free radical scavenging activity
A_negative control_ = negative control absorbance
A_sample_ = sample absorbance

### 2.10. In Vitro Determination of Sun Protection Factor (SPF)

SPF was determined according to an in vitro method [46], known for being practical and presenting a good correlation with in vivo methodologies. Thus, formulations were prepared using polawax^®^ cream as a cosmetic base and extracted samples from all months harvested of both species, separately, were incorporated at a concentration of 10%. For spectrophotometric analyses, liquid samples of each formulation were prepared at 0.2 mg/mL, using absolute ethanol as solvent. Subsequently, a scan was made between the wavelengths of 200–400 nm using a spectrophotometer UV-Vis, UV-2550 Shimadzu, with a 1 cm optical path length quartz cell, analyses was done in triplicate and absolute ethanol was also used as blank. SPF was calculated by Equation (2), and an assay was made in triplicate.


SPF = CF × Σ320−290 × EE(λ) × I(λ) × ABS(λ)
(2)

Equation (2) SPF formula.

where,

CF = 10 (correction factor)
EE(λ) = erythematogenic effect
I(λ) = Sun intensity
ABS(λ) = absorbance

### 2.11. Statistical Analyses

The data were obtained in triplicate, calculating the mean, standard error, and relative standard error. The statistical analysis was performed by comparison established through the analysis of variance (ANOVA one way), where the results were considered statistically different when *p* < 0.05, the level of significance adopted was 95% and also the post-test Tukey, using Graphpad Prism 6.01 software, San Diego, CA, USA. The calibration curves and correlation coefficients (r) were obtained and calculated by linear regression using Excel^®^ 2010. Principal component analysis (PCA) was used to obtain the correlation between the different data sets and a more distinct view of the relationship between the variables, as well as the variability of antioxidant activity and SPF. This analysis was performed with the Orange statistics 3.4 program.

## 3. Results

### 3.1. Seasonality Effects on Polyphenols, Flavonoids, and RA Content

**Figure 1:**
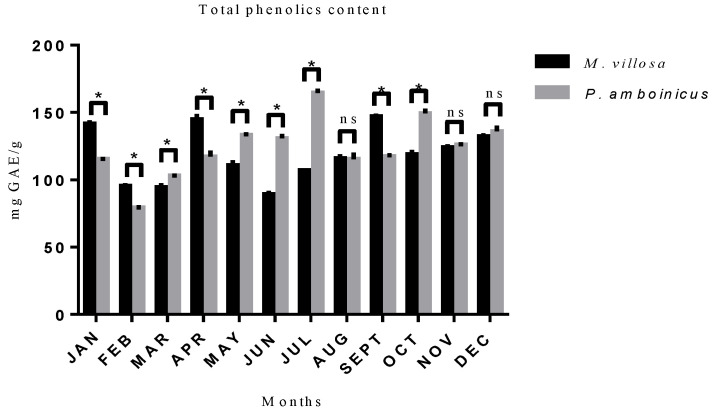
Polyphenols content of *M. villosa* and *P. amboinicus* throughout 12 months and their statistical differences. ns = nonsignificant. * statistical difference.

In a seasonal analysis of polyphenol content between *M. villosa* and *P. amboinicus*, statistical difference (*p* < 0.05) was found between the two species in every month of the year, except in August, November, and December, as shown in Figure 1. The best month for *M. villosa* harvest producing the highest total polyphenols content was September (147 mg GAE/g of crude extract). In *P. amboinicus* case, it was observed that the best month for its harvest was July (164.7 mg GAE/g of crude extract).

When the two species were evaluated individually, it was possible to observe that there was no statistical difference in total polyphenols content in several months of different seasons of the year in both *M. villosa* and *P. amboinicus*, showing that their production does not obey a specific trend for each season, e.g., January, which belongs to summer season did not show statistical difference (*p* > 0.05) when compared to April (autumn), August (winter), or October (spring). Thus, these results added to the fact that there are no well-defined seasons at the region where plants were collected, justified by their geographical location close to the equator line, seasons seem to have less influence in the variation of these metabolites concentration. Similar results were observed in a study by Woźniak et al. [47], where the concentrations of most polyphenols remained constant during different seasons, showing that they did not influence the variation of these metabolites.

**Figure 2:**
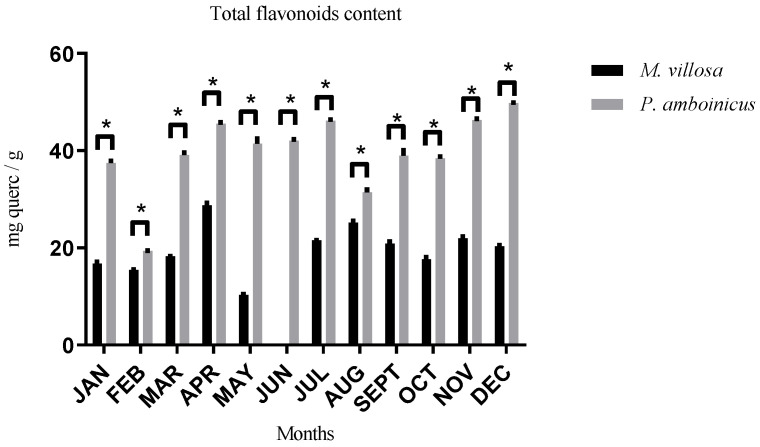
Flavonoids content of *M. villosa* and *P. amboinicus* throughout 12 months and their statistical differences. * statistical difference.

In the evaluation of flavonoids content, statistical difference (*p* < 0.05) was found among every month of the year between *M. villosa* and *P. amboinicus,* as shown in Figure 2. It was also observed that *P. amboinicus* presented the highest values of these metabolites during the whole year, when compared to the other studied species. The best month for *M. villosa* harvest was April (28.72 mg querc/g of crude extract) and for *P. amboinicus,* December (49.82 mg querc/g of crude extract).

As observed for total polyphenols content, when the two species are evaluated individually, it was seen that there was no statistical difference of flavonoids concentration in several months of different seasons in *M. villosa* and *P. amboinicus*, indicating that their production also did not obey a specific trend to each season, which shows that seasons seem to have less influence in variation of flavonoids quantitatively production.

**Figure 3:**
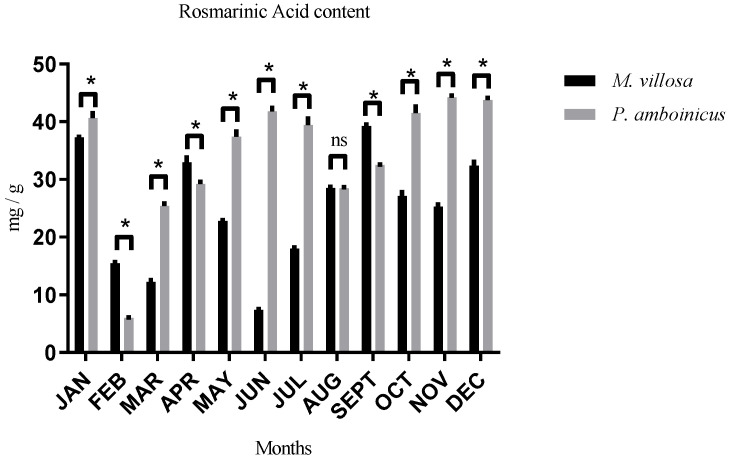
RA content of *M. villosa* and *P. amboinicus* extracts throughout 12 months and their statistical differences. ns = nonsignificant. * statistical difference.

According to a previous study by Terto et al. [40] and unpublished data [41], it was seen that RA was the most produced compound in CEE of aerial parts of *M. villosa* and *P. amboinicus*, and so because of this, its variation was also evaluated. Thus, it was observed that *P. amboinicus* had the highest concentrations of this metabolite in a good part of the year and the only month that there was no statistical difference in RA content between the two species was August, when they showed statistically equal production, as shown in Figure 3. The best month for *M. villosa* harvest was September (39.28 mg/g) and to *P. amboinicus*, it was observed that in six months of the year, RA concentrations remained practically constant, and it did not show statistical difference among them. These months were January, May, June, July, October, November, and December. Absolute values ranged from 39.46 to 44.24 mg/g, tables containing values of polyphenols, flavonoids and RA content of all months can be found in Supplementary Material.

As observed to polyphenols and flavonoids content, when the two plants are evaluated individually, no statistical difference was found in RA concentrations in several months of different seasons of the year, indicating that seasons seem to have less influence on RA content variation in both species, although *P. amboinicus* has shown to be more stable in this metabolite production.

Thus, according to obtained results, it is possible to observe that other factors may have a better influence on the production of polyphenols, flavonoids and RA than only seasons of the year, and it was possible to conclude that the best month to harvest *M. villosa* is September and July for *P. amboinicus*.

### 3.2. Seasonality Effects on Antioxidant Activity

**Figure 4:**
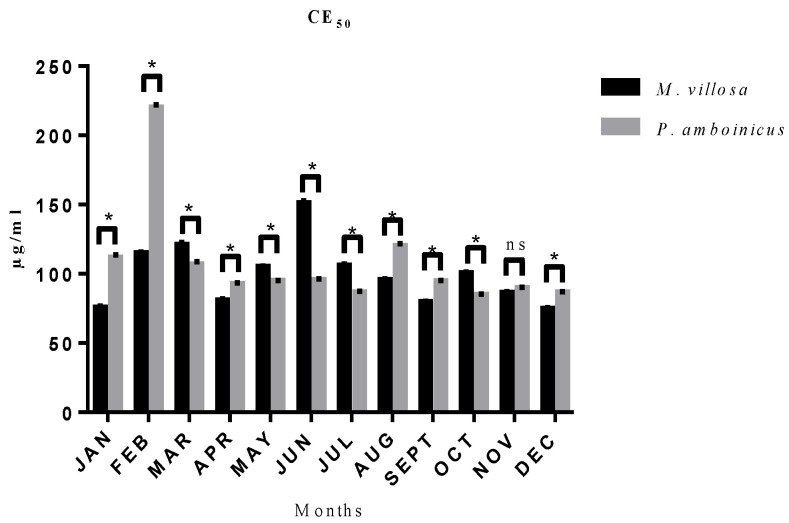
CEE of *M. villosa* and *P. amboinicus* EC_50_ in µg/mL and their statistical comparations throughout 12 months. ns = nonsignificant. * statistical difference.

Samples antioxidant activities were evaluated by DPPH test, and results were expressed as EC_50_. From Figure 4, a statistical difference is seen among all months, except for November, when samples of both plants showed statistically similar EC_50_. The month in which *M. villosa* extract showed better action against DPPH radicals is December (75.09 µg/mL) and for *P. amboinicus* is October (85.04 µg/mL), other results can be found in Supplementary Material.

### 3.3. Seasonality Effects on SPF

**Figure 5:**
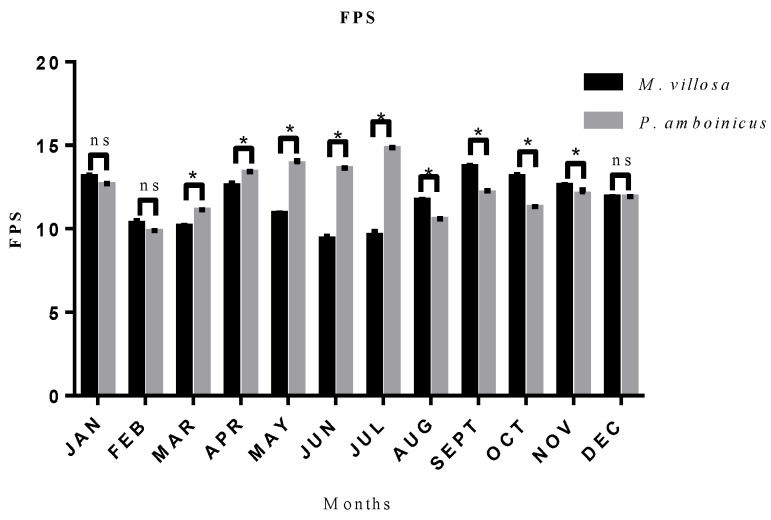
SPF of CEE of *M. villosa* and *P. amboinicus* and their statistical comparations throughout 12 months. ns = nonsignificant. * statistical difference.

From Figure 5, it was possible to evaluate monthly variations occurred on SPF. It was observed that January, February and December did not presented statistical difference between the two species, while in the rest of the year, this difference is significant. The best month for *M. villosa* harvest aiming the highest value of SPF was September (SPF = 13.73). Regarding *P. amboinicus*, it was observed that April, May, June and July showed the best values of SPF, what may indicate that autumn favors photoprotective activity of this plant, and SPF reaches its maximum of 14.79, other results can also be found in Supplementary Material. 

When is considered that in this test were only used plant extracts of the two studied species incorporated at 10% in Polawax^®^ cream, it is possible to suggest that these results are promising in the photoprotection area, since reaching SPF levels close to 15 without the addition of any synthetic sunscreen is not easy, as shows the study made by Oliveira et al. [48], that evaluated the SPF of *Schinus terebinthifolius* Raddi ethanolic extracts and none formulation composed only but extracts had SPF above 5.08. In another study made by Mota et al. [49], it was evaluated SPF of *Psidium guajava* ethanolic extract and they found SPF = 1. Similarly, Mota et al. [50] evaluated SPF of *Nephelium lappaceum* L. ethanolic extract (peels) and its SPF = 0.4.

Thus, the two studied plants show significant values of SPF throughout the year, which are higher than what is required by the Brazilian Regulatory Agency (ANVISA) in its Resolution of the Collegiate Board of Directors of June, the 30th (2012) [51], that determines that only photoprotective formulations with SPF ≥6 are valid.

### 3.4. Principal Components Analysis (PCA)

To assess the relationship among all existing variables, multivariate analysis of main components was used, which made it possible to observe several correlations between the concentration of RA, phenolic and total flavonoid content, SPF and EC_50_, as well as the relationship of these variables with the rainfall index and solar radiation during the year.

**Figure 6:**
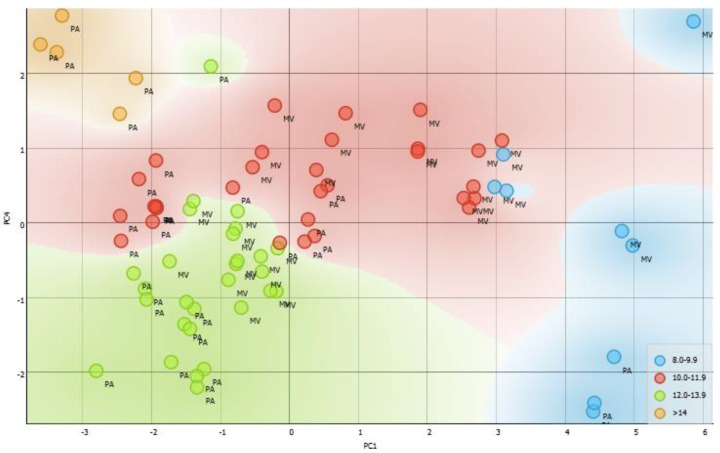
Principal components analysis showing correlation of groups of PC4 versus PC1 using obtained data from the analysis of phenolic and total flavonoid content, RA content, EC_50_, SPF, the incidence of solar radiation, and level of precipitation.

As shown in Figure 6, the correlation between PC4 × PC1 of the PCA explained 80% of the existing groups. For a better understanding of the results, SPF values were categorized through colors to differentiate the value of this parameter in each species. In this figure, it is possible to observe that samples that showed the highest SPF values were grouped more to the left in green, for both *M. villosa* and *P. amboinicus*, in addition, it is possible to observe that something beyond the SPF influence in these groups and it is the phenolic content.

**Figure 7:**
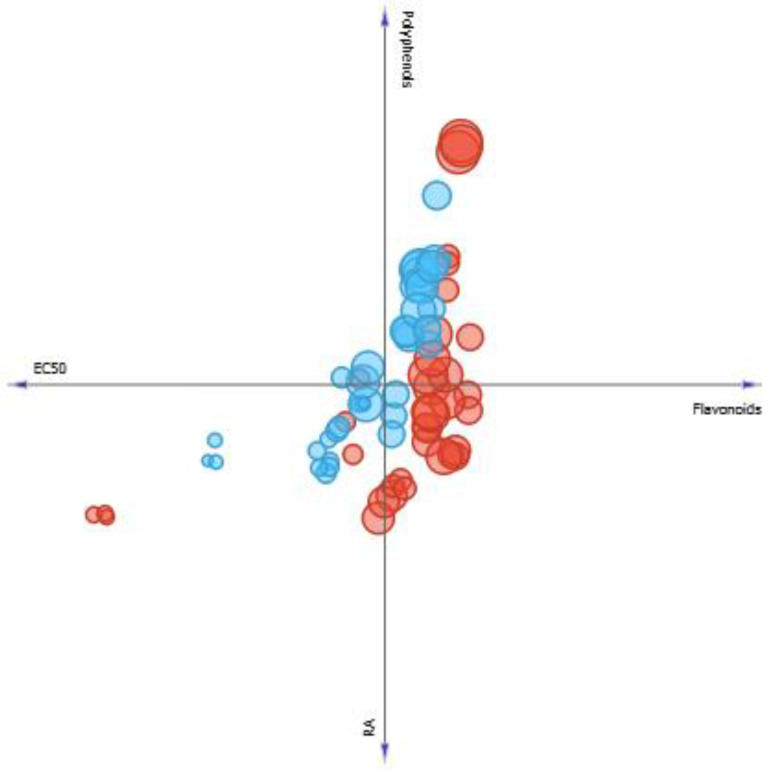
Four axes linear projection correlating the content of phenolics, flavonoids, rosmarinic acid, EC_50_ of *M. villosa* (blue circles) *and P. amboinicus* (red circles). Circles size correspond to a greater or lesser SPF value found.

According to the linear projection in four axes represented in Figure 7, it is possible to observe blue and red circles, that represents *M. villosa* (MV) and *P. amboinicus* (PA), respectively. Larger and smaller circles represent higher or lower SPF values. Thus, it is seen that the greater the amount of phenolics and flavonoids, the greater the diameter of the blue circles and, consequently, the greater the SPF of *M. villosa* extracts. It is also observed that RA presence favors a lower EC_50_, indicating better antioxidant activity, when it occurs.

When it comes to *P. amboinicus*, in general, it is seen that flavonoids and RA presence keep SPF values similar and low polyphenols concentrations do not favor this factor as much, however, when they reach the maximum concentration found in the species, the highest SPF (14.79) value observed in the present study was found.

**Figure 8:**
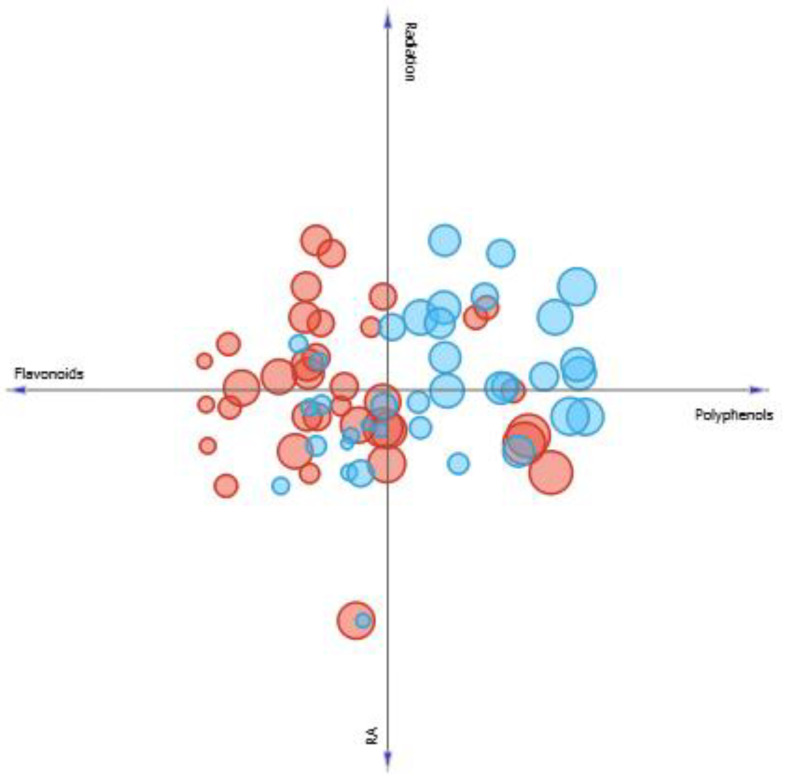
Linear projection correlating the content of phenolics, flavonoids and rosmarinic acid of *M. villosa* (blue circles) *and P. amboinicus* (red circles) to solar radiation. Circles size correspond to a greater or lesser SPF value found.

Another parameter observed was the influence of the solar radiation on the concentration of studied secondary metabolites and its consequences to SPF. Thus, evaluating the linear projection presented in Figure 8, it is seen that the solar radiation has great influence on increasing or decreasing SPF in *M. villosa.* In months when both solar radiation and polyphenols production are higher, SPF of *M. villosa* extracts increases. However, in months when production of flavonoids and RA is high, but the level of solar radiation is lower, SPF is also lower, suggesting that production of specific components with photoprotective properties is stimulated by solar radiation.

These results are corroborated by Dolzhenko et al. [52], who evaluated the influence of UVB radiation on polyphenols production in *Mentha x piperita* L. species. In their study, it was seen that polyphenols content increased after irradiation of the plant with UVB radiation, and it stimulated the production of flavonoids with light-absorbing properties at the wavelength corresponding to UVB radiation, such as eriocitrin, hesperidin, and kaempferol 7-O-rutinoside, and it also caused production decrease of narirutin, 4′-methoxykaempferol 7-O-rutinoside, suggesting a possible transformation of these latest flavonoids cited in those with increased production. The same study also showed that there is a decrease in essential oils production in *M. piperita* and an increase on polyphenols production and suggests a correlation between these results, showing that UVB radiation may favor one biosynthetic path over another.

In *P. amboinicus* samples, different observations were made. Through Figure 8, it is seen that the solar radiation does not seem to have an influence as directed as in *M. villosa*, because although SPF was high in samples when solar radiation was higher compared to others, the same observation was made when RA and flavonoids concentrations are higher, and the sample with the highest SPF is found when polyphenols content is higher, but radiation level is lower.

In a study by Takshak et al. [53], it was seen that supplementation of UVB radiation in *Coleus forskohlii* (or *P. barbatus*) favored phenylpropanoid paths generating production of flavonoids, for example. On the other hand, it was seen on the same study, that UVB radiation also induced an increase of carotenoids production in the leaves of this species, such as lycopene and β-carotene for its protection. So, although it is necessary more studies to confirm UV radiation role in secondary metabolites production in *P. amboinicus*, it is possible to suggest that another biosynthetic path may be favored to increase its SPF.

**Figure 9:**
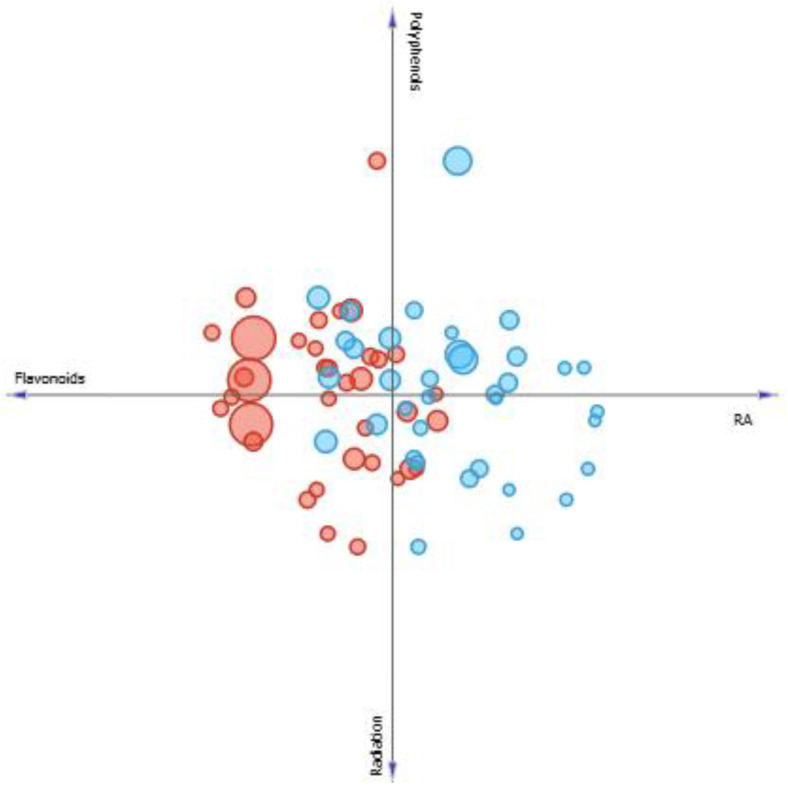
Linear projection correlating the content of phenolics, flavonoids, and rosmarinic acid of *M. villosa* (blue circles) *and P. amboinicus* (red circles) to solar radiation. Circles size corresponds to a greater or lesser EC_50_ value found.

On the other hand, it is seen in Figure 9, that radiation is an important factor in *P. amboinicus* antioxidant activity since it is showed that when solar radiation levels are higher, EC_50_ (represented by sample circles size) decreases, which means a better antioxidant activity. This result is corroborated with both Takshak et al. [53] and Takshak et al. [54] that showed an increase of antioxidant activity of *Coleus forskohlii* extracts when it was supplemented by UVB radiation. A similar result was observed to *M. villosa* through Figure 9.

In addition, it is also important to highlight that as seen in a review made by Gobbo-neto and Lopes [8], certain polyphenols may have their production increased, as in the case of *Marchantia polymorpha* in which the proportion of luteolin glycosides/apigenin glycosides increases by the influence of exposure to UVB radiation. In this situation, although the absorption capacity of UVB rays does not increase, because luteolin derivates are more efficient in disperse observed energy, it causes an increase of defense antioxidant levels in the plant. The same observation was made in the flavonoids/hydroxycinnamates ratio, with an increase in this proportion being seen by induction of UVB radiation. So, it is seen that the ability to act as an antioxidant and not only as an absorber of UVB radiation, it is important to define which metabolites will be produced to act against UVB rays. Thus, more studies need to be done to better understand the metabolic changes mainly in *P. amboinicus.*

**Figure 10:**
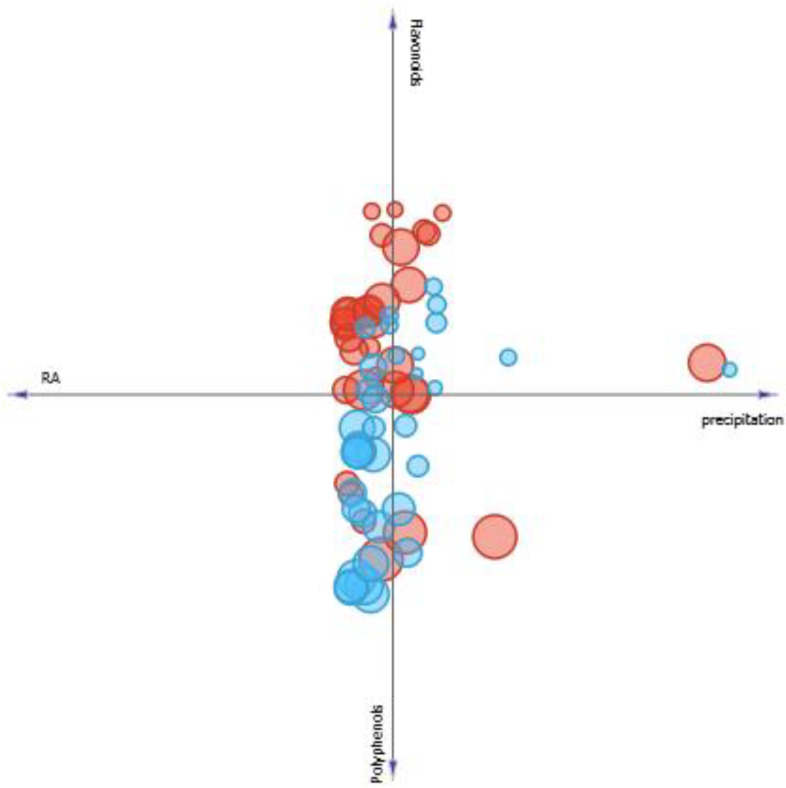
Linear projection correlating the content of phenolics, flavonoids, and rosmarinic acid of *M. villosa* (blue circles) *and P. amboinicus* (red circles) to precipitation. Circles size correspond to a greater or lesser SPF value found.

**Figure 11:**
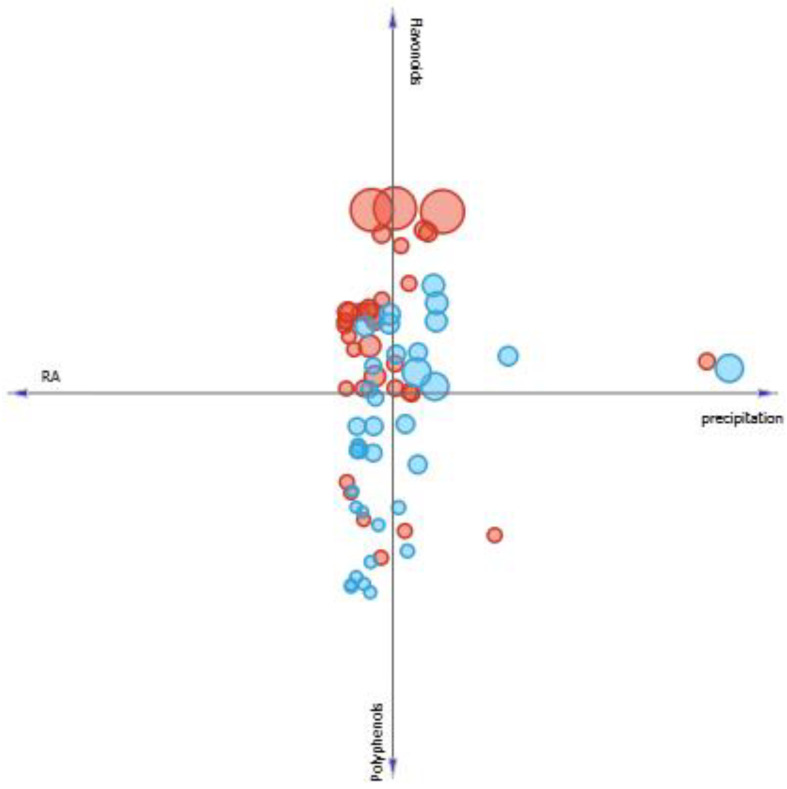
Linear projection correlating the content of phenolics, flavonoids, and rosmarinic acid of *M. villosa* (blue circles) *and P. amboinicus* (red circles) to precipitation. Circles size corresponds to a greater or lesser EC_50_ value found.

In the case of precipitation levels, it was observed in Figure 10, that in general, it does not favor both SPF and antioxidant activity increase (Figure 11) of extracts of both plants studied, since the lower precipitation levels, the higher SPF and lower EC_50_, indicating a better antioxidant activity.

According to Gobbo-neto and Lopes [8], there are controversies about what can occur in polyphenol production depending on environmental levels of precipitation, showing that its production can be increased or decreased, with no trend or pattern to be followed, suggesting that there is no clear correlation between these metabolites concentrations and hydric stress, but short periods of dryness can lead to an increase of phenolic compounds production. Studies such as Gomes et al. [55] showed a predominance of flavonoids during summer in *Lippia alba* leaves. A similar result was found by Ribeiro et al. [1], where it was found that the highest concentration of total phenolic compounds in the inner bark of *Secondatia floribunda* A. DC. during the dry season.

Thus, biological activities such as antioxidant activity can be affected since several studies confirm a direct correlation between this activity with phenolic compounds. Because of that, more studies are necessary to establish a better knowledge about what occurs in *M. villosa* and *P. amboinicus* metabolism.

## 4. Conclusions

In agreement with all obtained results, it was possible to conclude that, in general, seasonality did not directly affect polyphenols, flavonoids, and RA quantitative production in both species. The best month to harvest *M. villosa* was September and *P. amboinicus* was July. It could also be concluded that higher concentrations of polyphenols and flavonoids favor the increase of SPF in *M. villosa* and concentration of RA influences the antioxidant activity of this species, while in *P. amboinius*, the presence of flavonoids and RA maintained SPF values similar and when the phenolic compounds reached the maximum concentration in this species, the highest SPF value of the study was found (SPF = 14.79). Moreover, it was observed that solar radiation has a clearer influence in phenolic compound production and in the increase of SPF in *M. villosa* than in *P. amboinicus,* and it favors the improvement of antioxidant activities of both species. In the case of precipitation, it does not seem to favor either photoprotective or antioxidant activities of both species studied.

## Data Availability

The data presented in this study are available in Supplementary Material.

## Supplementary Materials

The following are available online at https://www.mdpi.com/1999-4923/13/1/110/s1, Figure S1: Calibration curve of Quercetin, Figure S2: Calibration curve of Gallic acid standard, Figure S3: Rosmarinic acid stardand linear regression for its quantification in *M. x villosa* and *P. amboinicus*, Figure S4: Superimposed chromatograms of rosmarinic acid triplicate (retention times at 18.24; 17.65; 17.69 min) for its quantification in January, *Mentha x villosa*, Figure S5: Superimposed chromatograms of rosmarinic acid triplicate (retention times approx-imately at 17.60 min) for its quantification in February, *Mentha x villosa*, Figure S6: Superimposed chromatograms of rosmarinic acid triplicate (retention times approx-imately at 17.60 min) for its quantification in March, *Mentha x villosa*, Figure S7: Superimposed chromatograms of rosmarinic acid triplicate (retention times at 17.65; 17.53; 17.52 min) for its quantification in April, *Mentha x villosa*, Figure S8: Superimposed chromatograms of rosmarinic acid triplicate (retention times approx-imately at 17.60 min) for its quantification in May, *Mentha x villosa*, Figure S9: Superimposed chromatograms of rosmarinic acid triplicate (retention times approx-imately at 17.53 min) for its quantification in June, *Mentha x villosa*, Figure S10: Superimposed chromatograms of rosmarinic acid triplicate (retention times approx-imately at 17.60 min) for its quantification in July, *Mentha x villosa*, Figure S11: Superimposed chromatograms of rosmarinic acid triplicate (retention times approximately at 17.60 min) for its quantification in August, *Mentha x villosa*, Figure S12: Superimposed chromatograms of rosmarinic acid triplicate (retention times approx-imately at 17.60 min) for its quantification in September, *Mentha x villosa*, Figure S13: Superimposed chromatograms of rosmarinic acid triplicate (retention times approx-imately at 17.63 min) for its quantification in October, *Mentha x villosa*, Figure S14: Superimposed chromatograms of rosmarinic acid triplicate (retention times approx-imately at 17.72 min) for its quantification in November, *Mentha x villosa*, Figure S15: Superimposed chromatograms of rosmarinic acid triplicate (retention times at 17.72; 17.71; 17.92 min) for its quantification in December, *Mentha x villosa*, Figure S16: Superimposed chromatograms of rosmarinic acid triplicate (retention times at 10.07; 9.54; 10.07 min) for its quantification in January, Pectranthus amboinicus, Figure S17: Superimposed chromatograms of rosmarinic acid triplicate (retention times approx-imately at 10.14 min) for its quantification in February, Pectranthus amboinicus, Figure S18: Superimposed chromatograms of rosmarinic acid triplicate (retention times approx-imately at 9.55 min) for its quantification in March, Pectranthus amboinicus, Figure S19: Superimposed chromatograms of rosmarinic acid triplicate (retention times approx-imately at 9.56 min) for its quantification in April, Pectranthus amboinicus, Figure S20: Superimposed chromatograms of rosmarinic acid triplicate (retention times approx-imately at 9.57 min) for its quantification in June, Pectranthus amboinicus, Figure S21: Superimposed chromatograms of rosmarinic acid triplicate (retention times at 9.60; 9.62; 10.15 min) for its quantification in July, Pectranthus amboinicus, Figure S22: Superimposed chromatograms of rosmarinic acid triplicate (retention times approx-imately at 10.14 min) for its quantification in August, Pectranthus amboinicus, Figure S23: Superimposed chromatograms of rosmarinic acid triplicate (retention times at 9.64; 10.19; 10.19 min) for its quantification in October, Pectranthus amboinicus, Figure S24: Superimposed chromatograms of rosmarinic acid triplicate (retention times at 10.05; 9.64; 9.61 min) for its quantification in November, Pectranthus amboinicus, Figure S25: Superimposed chromatograms of rosmarinic acid triplicate (retention times approx-imately at 9.65 min) for its quantification in December, Pectranthus amboinicus, Table S1: Results of month-to-month quantification of rosmarinic acid in mg/g, *Mentha x villosa*, Table S2: Results of month-to-month quantification of rosmarinic acid in mg/g, *Plectranthus amboinicus,* Table S3: SPF results month by month, *Mentha x villosa*, Table S4: SPF results month by month, *Plectranthus amboinicus*, Table S5: Results of total phenolic content month by month mg GAE/ g, *Mentha x villosa*, Table S6: Results of the total phenolic content month by month mg GAE/g, *Plectranthus am-boinicus*, Table S7: Results of total flavonoid content month by month mg querc/ g, *Mentha x villosa*, Table S8: Results of total flavonoid content month by month mg querc/ g, *Plectranthus amboinicus*, Table S9: Radiation data used (kJ/m²), Table S10. Precipitation levels used (mm³).
