# Supplementary material for: Seasonal Variations of Polyphenols Content, Sun Protection Factor and Antioxidant Activity of Two Lamiaceae Species
Source: Pharmaceutics. 2021 Jan 16;13(1):110. doi: 10.3390/pharmaceutics13010110 (PMC7829895; doi:10.3390/pharmaceutics13010110)
Supplement: Supplementary file 1 [file pharmaceutics-13-00110-s001.pdf]

pharmaceutics

MDPI

Supplementary Materials: Seasonal Variations of Polyphenols Content, Sun Protection Factor and Antioxidant Activity of Two Lamiaceae Species

Juliana de Medeiros Gomes, Márcio Vinícius Cahino Terto, Sócrates Golzio do Santos, Marcelo Sobral da Silva and Josean Fechine Tavares *

Table S1. Results of month-to-month quantification of rosmarinic acid in mg / g, *Mentha x villosa.*

Months	Jan	Feb	Mar	Apr	May	Jun	Jul	Aug	Setp	Oct	Nov	Dec
	37.16	14.85	12.4	31.38	22.73	7.66	17.5	28.89	39.7	26.18	24.52	32.97
**Triplicate**	37.17	15.64	11.55	34.11	23.05	7.12	18.04	28.38	38.92	28.46	25.46	31.1
	37.52	15.76	12.45	33.36	22.52	7.29	18.4	28.29	39.23	26.66	25.81	33.04
**Mean**	37.29	15.42	12.21	32.95	22.77	7.35	17.98	28.52	39.28	27.1	25.26	32.37
**Error**	0.21	0.49	0.58	1.41	0.27	0.28	0.45	0.32	0.39	1.2	0.67	1.1
**Relative error**	0.56	3.21	4.77	4.28	1.17	3.74	2.5	1.14	1	4.44	2.64	3.39

Table S2. Results of month-to-month quantification of rosmarinic acid in mg / g, *Plectranthus amboinicus.*

Months	Jan	Feb	Mar	Apr	May	Jun	Jul	Aug	Setp	Oct	Nov	Dec
	39.3	6.03	24.52	29.1	35.65	41.09	37.79	28.33	32.37	41.82	44.65	42.91
**Triplicate**	42.22	6.12	25.82	28.65	38.66	41.5	41.44	28.08	32.55	43.2	44.38	44.12
	40.4	5.86	25.83	29.95	37.92	42.9	39.02	28.86	32.54	39.52	43.7	44.18
**Mean**	40.64	6	25.39	29.23	37.41	41.83	39.46	28.43	32.49	41.51	44.24	43.73
**Error**	1.47	0.14	0.76	0.66	1.57	0.95	1.92	0.4	0.1	1.86	0.49	0.71
**Relative error**	3.63	2.25	2.98	2.26	4.19	2.27	4.87	1.42	0.32	4.48	1.11	1.63

Table S3. SPF results month by month, *Mentha x villosa.*

Months	Jan	Feb	Mar	Apr	May	Jun	Jul	Aug	Setp	Oct	Nov	Dec
	13.05	10	10.08	12.2	10.81	9.03	9.15	11.65	13.6	13.26	12.52	11.82
**Triplicate**	12.94	10.58	10.14	12.7	10.93	9.54	9.81	11.72	13.78	13.2	12.61	11.91
	13.31	10.38	10.23	12.77	10.96	9.55	9.87	11.75	13.81	12.93	12.67	11.93
**Mean**	13.1	10.32	10.15	12.55	10.9	9.38	9.61	11.71	13.73	13.13	12.6	11.89
**Error**	0.19	0.29	0.08	0.31	0.08	0.3	0.4	0.05	0.12	0.18	0.07	0.06
**Relative error**	1.45	2.81	0.75	2.5	0.71	3.15	4.15	0.46	0.84	1.34	0.59	0.52

Table S4. SPF results month by month, *Plectranthus amboinicus.*

Months	Jan	Feb	Mar	Apr	May	Jun	Jul	Aug	Setp	Oct	Nov	Dec
Triplicate	12.58	9.77	11.04	13.28	13.92	13.52	14.67	10.5	11.89	11.19	11.78	11.81
	12.26	9.85	11.12	13.4	14.18	13.62	14.82	10.56	12.25	11.3	11.92	11.91
	12.6	9.91	11.15	13.45	13.52	13.65	14.88	10.61	12.3	11.35	12.55	11.93
**Mean**	12.65	9.84	11.1	13.37	13.88	13.6	14.79	10.56	12.15	11.28	12.08	11.89
**Error**	0.1	0.07	0.05	0.09	0.33	0.07	0.11	0.05	0.22	0.08	0.41	0.06
**Relative error**	0.8	0.73	0.5	0.67	2.4	0.5	0.75	0.5	1.85	0.74	3.4	0.52

Table S5. Results of total phenolic content month by month mg GAE/ g, *Mentha x villosa.*

Months	Jan	Feb	Mar	Apr	May	Jun	Jul	Aug	Setp	Oct	Nov	Dec
Triplicate	142.76	95.87	96.72	149.7	115.48	88.74	106.66	116.26	147.61	115.17	123.04	130.44
	142.65	94.94	91.6	143.27	108.31	88	107.11	113.16	146.06	120.6	124.63	133.54
	139.7	95.94	94.9	141.721	108.27	91.22	107.42	118.7	147.42	120.79	125.13	132.57
**Mean**	141.7	95.58	94.41	144.9	110.69	89.32	107.06	116.04	147.03	118.85	124.27	132.19
**Error**	1.73	0.56	2.59	4.23	4.15	1.68	0.38	2.78	0.84	0.64	1.09	1.59
**Relative error**	1.22	0.59	2.75	2.92	3.75	1.89	0.35	2.39	0.57	0.54	0.88	1.2

Table S6. Results of the total phenolic content month by month mg GAE/g, *Plectranthus amboinicus.*

Months	Jan	Feb	Mar	Apr	May	Jun	Jul	Aug	Setp	Oct	Nov	Dec
Triplicate	115.44	79.16	102.84	118.5	134.51	129.01	163.12	114.86	118.19	146.88	126.49	141.33
	115.21	79.59	103.31	121.8	131.76	129.16	167.54	110.07	116.26	152.96	126.06	134.43
	115.64	79.47	103	111.6	133.31	134.36	163.46	121.41	118.19	148.31	126.18	132.3
**Mean**	115.43	79.41	103.05	117.3	133.19	130.84	164.7	115.43	117.55	149.38	126.24	136.02
**Error**	0.21	0.22	0.24	5.2	1.38	3.04	2.46	5.72	1.12	3.18	0.22	4.72
**Relative error**	0.18	0.28	0.23	4.43	1.03	2.33	1.49	4.95	0.95	2.13	0.17	3.47

Table S7. Results of total flavonoid content month by month mg querc/ g, *Mentha x villosa.*

Months	Jan	Feb	Mar	Apr	May	Jun	Jul	Aug	Sept	Oct	Nov	Dec
Triplicate	16.21	15.73	18.26	29.44	10.49	Nd	21.49	24.52	20.06	16.92	21.38	20.76
	16.46	15.25	18.23	27.59	10.09	Nd	21.57	25.57	21.19	17.64	22.02	20.2
	17.51	15.33	18.4	29.12	10.41	Nd	21.57	25.49	21.35	18.37	22.59	20.03
**Mean**	16.73	15.44	18.3	28.72	10.33	Nd	21.54	25.2	20.87	17.64	22	20.33
**Error**	0.68	0.26	0.09	0.99	0.21	Nd	0.05	0.58	0.7	0.72	0.6	0.38
**Relative error**	4.1	1.68	0.47	3.45	2.06	Nd	0.22	2.32	3.37	4.11	2.75	1.87

*Nd = Not detected.

Table S8. Results of total flavonoid content month by month mg querc/ g, *Plectranthus amboinicus.*

Months	Jan	Feb	Mar	Apr	May	Jun	Jul	Aug	Sept	Oct	Nov	Dec
Triplicate	37.18	19.39	38.56	45.46	42.29	41.81	46.4	30.79	38.37	38.8	46.01	49.77
	37.1	19.22	39.68	45.87	40.2	42.37	46.08	31.51	38.21	38.23	46.4	49.77
	37.99	19.39	38.96	45.22	41.81	41.89	46.01	32	40.3	38.31	46.49	49.83
**Mean**	37.43	19.34	39.07	45.52	41.43	42.02	46.16	31.43	38.96	38.45	46.3	49.82
**Error**	0.49	0.09	0.57	0.32	1.1	0.3	0.21	0.61	1.17	0.3	0.26	0.09
**Relative error**	1.31	0.48	1.46	0.72	2.65	0.73	0.46	1.94	2.99	0.79	0.56	0.19

Table S9. EC_{50} results month by month in µg/ml, *Mentha x villosa*.

Months	Jan	Feb	Mar	Apr	May	Jun	Jul	Aug	Setp	Oct	Nov	Dec
Triplicate	76.47	114.78	117.96	80.7	104.29	153.76	105.31	94.99	79.85	99.96	85.18	75.73
	7446	116.11	122.15	79.89	106.14	150.1	105.99	97.09	79.59	101.88	86.59	73.88
	77.06	114.67	123.14	82.64	105.42	150.5	107.65	95.16	80.3	100.44	87.67	75.66
**Mean**	75.99	115.19	121.08	81.08	105.28	151.45	106.32	95.74	79.91	100.76	86.48	75.09
**Error**	1.37	0.8	2.75	1.41	0.93	2	1.2	1.17	0.36	1	1.25	1.04
**Relative error**	1.8	0.7	2.27	1.74	0.89	1.32	1.13	1.22	0.45	0.99	1.44	1.39

Table S10. EC_{50} results month by month in μg/ml, *Plectranthus amboinicus.*

Months	Jan	Feb	Mar	Apr	May	Jun	Jul	Aug	Setp	Oct	Nov	Dec
Triplicate	108.69	218,25	106.09	92.07	95.16	96.42	86.69	122.95	94.62	85.62	90.87	86.22
	114.57	223,83	110.5	93.38	95.43	96.07	86.7	120.28	93.94	84.62	89.49	87.3
	112.96	219.04	104.64	93.39	95.12	95.56	87.73	117.77	95.88	84.88	89.5	87.04
**Mean**	112.07	220.37	107.08	92.95	95.24	96.01	87.04	120.33	94.82	85.04	89.95	86.85
**Error**	3.04	3.02	3.05	0.76	0.17	0.43	0.6	2.59	0.99	0.52	0.79	0.56
**Relative error**	2.72	1.37	2.85	0.81	0.18	0.45	0.68	2.15	1.04	0.61	0.88	0.64

Table S11. Radiation data used (kJ/m^2).

Months	Jan	Feb	Mar	Apr	May	Jun	Jul	Aug	Setp	Oct	Nov	Dec
**1**	197641.060	203520.460	181576.322	196133.069	154980.228	173300.573	164603.583	182309.039	177154.311	224129.173	221421.193	260510.439
**2**	201835.671	163412.757	144502.035	160850.620	171977.400	80859.9140	150842.713	150422.667	177392.423	189475.618	205187.561	191501.764
**3**	224238.660	182924.830	211545.221	190800.970	184121.131	170916.486	168212.962	219538.095	238614.400	228766.061	260510.439	254396.450

Table S12. Precipitation levels used (mm^3).

Months	Jan	Feb	Mar	Apr	May	Jun	Jul	Aug	Setp	Oct	Nov	Dec
**1**	2	38.4	36.6	83	90.2	73.6	85.4	21.8	34	0.2	0.2	0.2
**2**	1.8	111.6	103.2	41	22.8	554.2	52.4	27.2	11.8	27.4	0	13.6
**3**	32.6	2.6	109.2	71.4	71	101	225	30.6	24	4	0	5.6

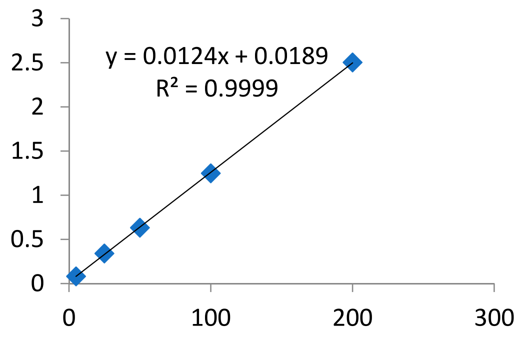

Figure S1. Calibration curve of Quercetin.

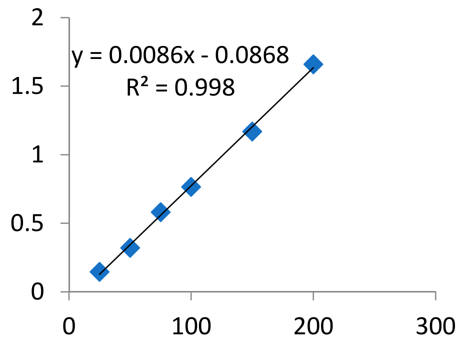

Figure S2. Calibration curve of Gallic acid standard.

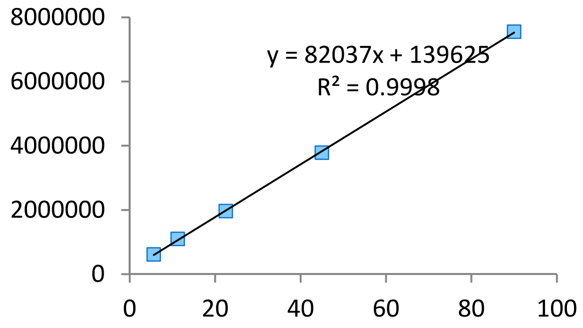

Figure S3. Rosmarinic acid stardand linear regression for its quantification in *M. x villosa* and *P. amboinicus*.

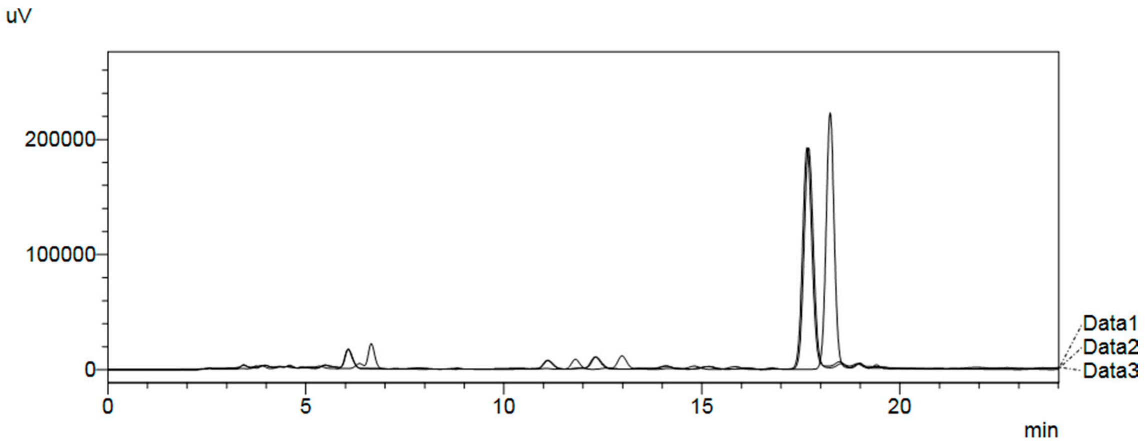

Figure S4. Superimposed chromatograms of rosmarinic acid triplicate (retention times at 18.24; 17.65; 17.69 min) for its quantification in January, *Mentha x villosa*.

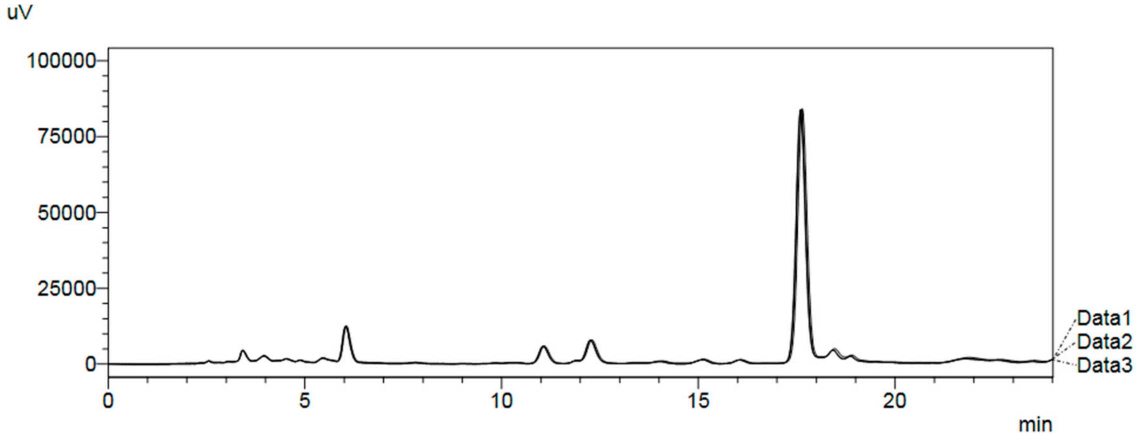

Figure S5. Superimposed chromatograms of rosmarinic acid triplicate (retention times approximately at 17.60 min) for its quantification in February, *Mentha x villosa*.

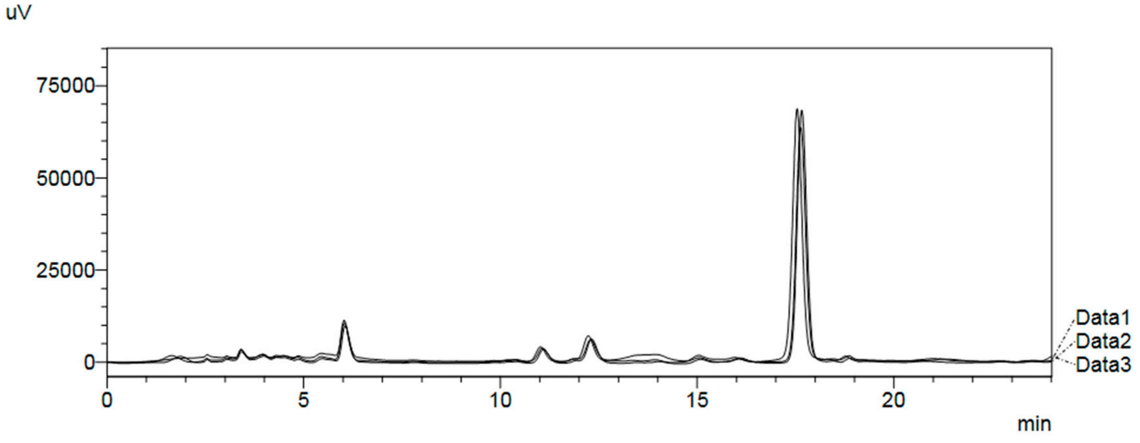

Figure S6. Superimposed chromatograms of rosmarinic acid triplicate (retention times approximately at 17.60 min) for its quantification in March, *Mentha x villosa*.

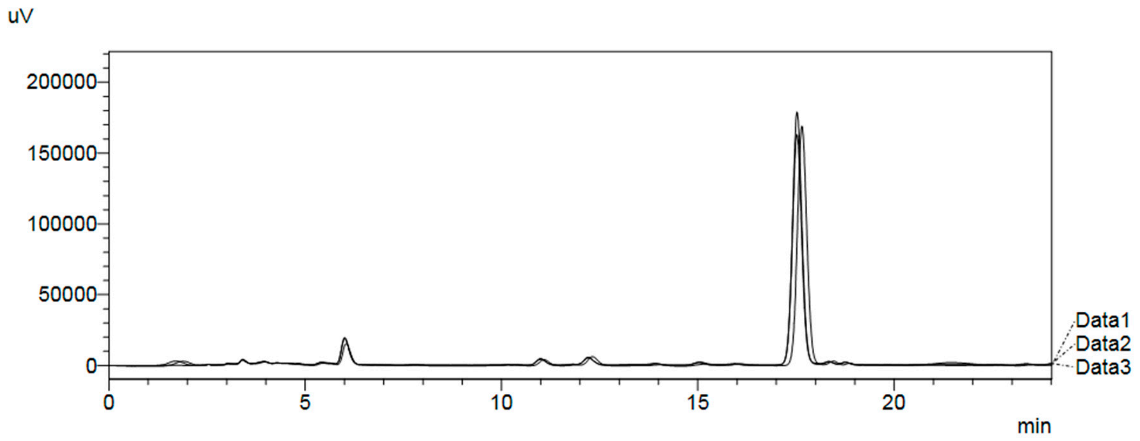

Figure S7. Superimposed chromatograms of rosmarinic acid triplicate (retention times at 17.65; 17.53; 17.52 min) for its quantification in April, *Mentha x villosa*.

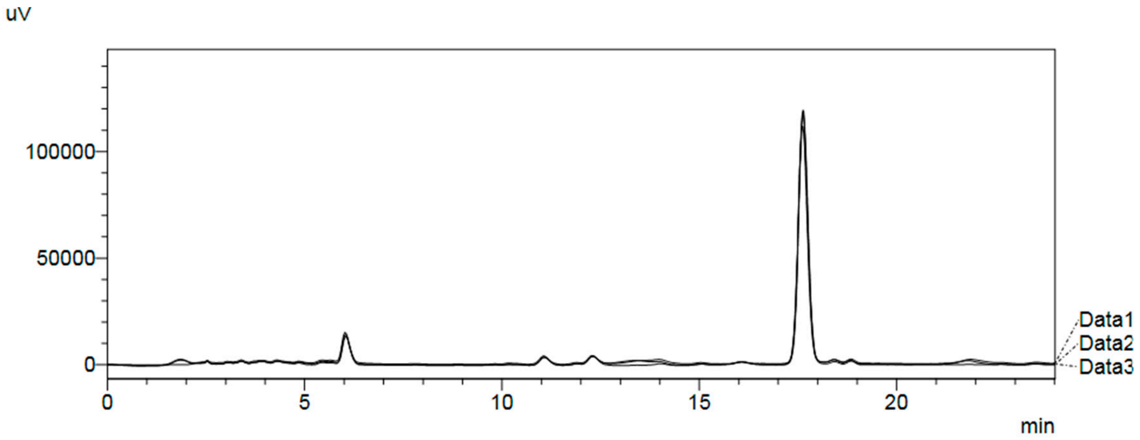

Figure S8. Superimposed chromatograms of rosmarinic acid triplicate (retention times approximately at 17.60 min) for its quantification in May, *Mentha x villosa*.

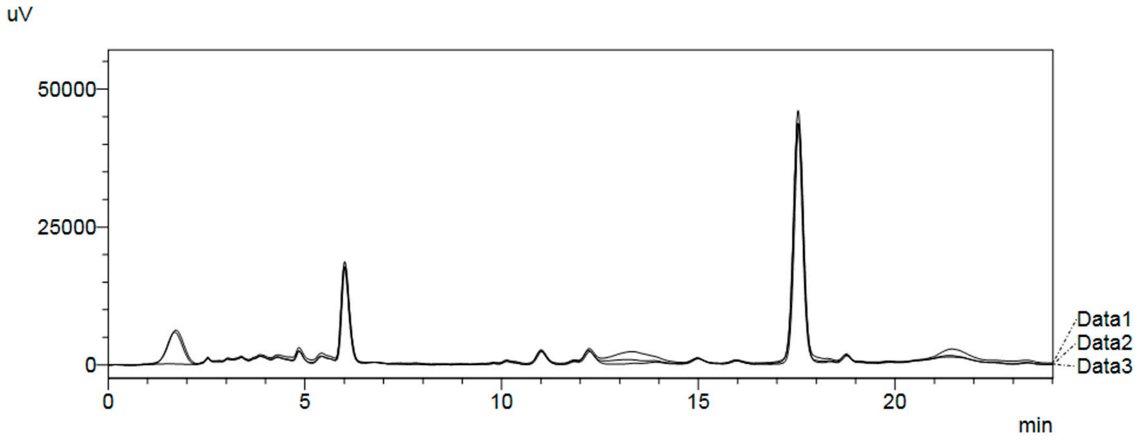

Figure S9. Superimposed chromatograms of rosmarinic acid triplicate (retention times approximately at 17.53 min) for its quantification in June, *Mentha x villosa*.

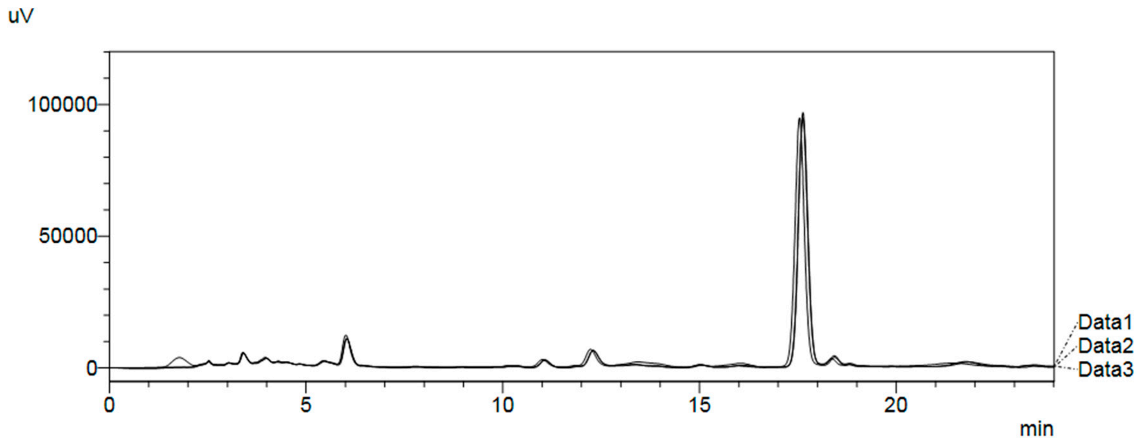

Figure S10. Superimposed chromatograms of rosmarinic acid triplicate (retention times approximately at 17.60 min) for its quantification in July, *Mentha x villosa*.

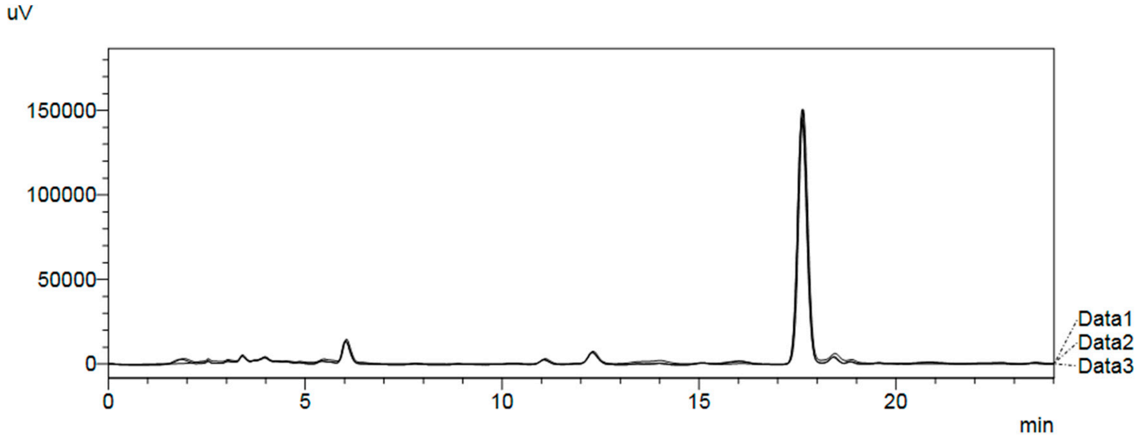

Figure S11. Superimposed chromatograms of rosmarinic acid triplicate (retention times approximately at 17.60 min) for its quantification in August, *Mentha x villosa*.

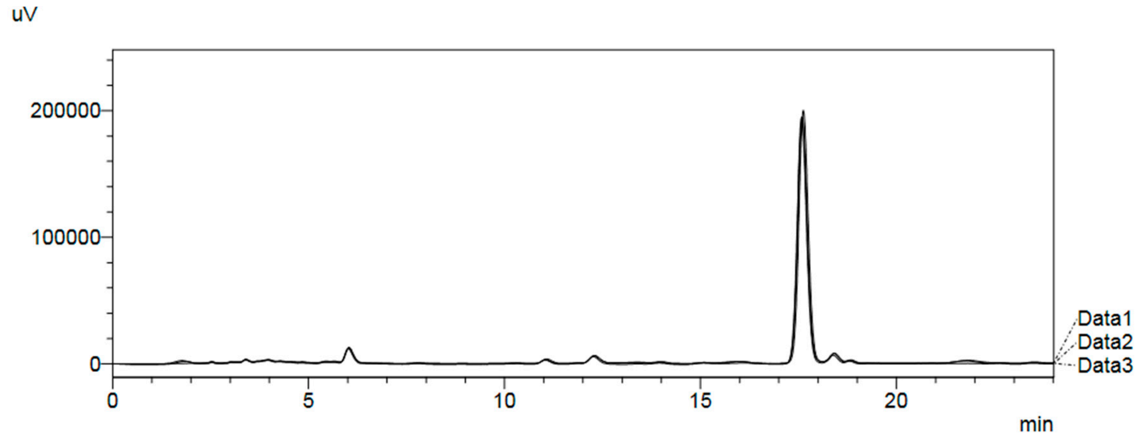

Figure S12. Superimposed chromatograms of rosmarinic acid triplicate (retention times approximately at 17.60 min) for its quantification in September, *Mentha x villosa*.

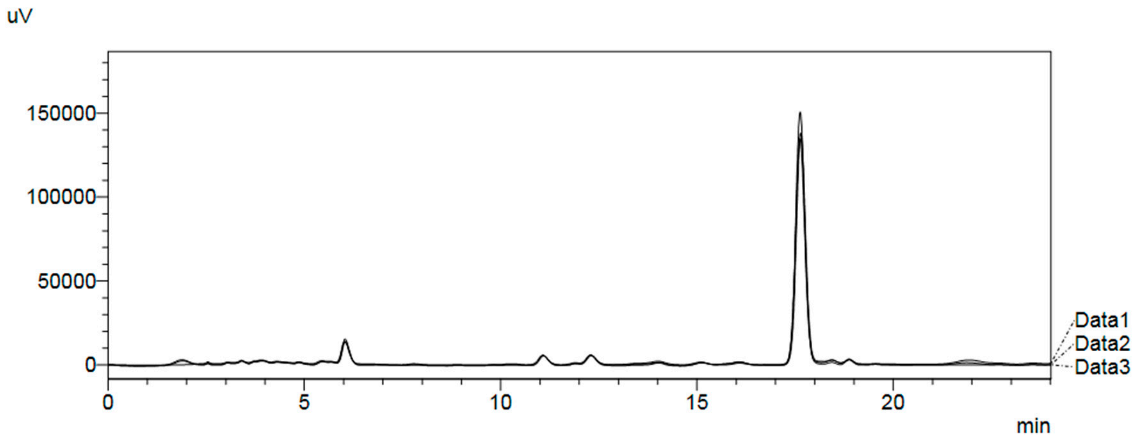

Figure S13. Superimposed chromatograms of rosmarinic acid triplicate (retention times approximately at 17.63 min) for its quantification in October, *Mentha x villosa*.

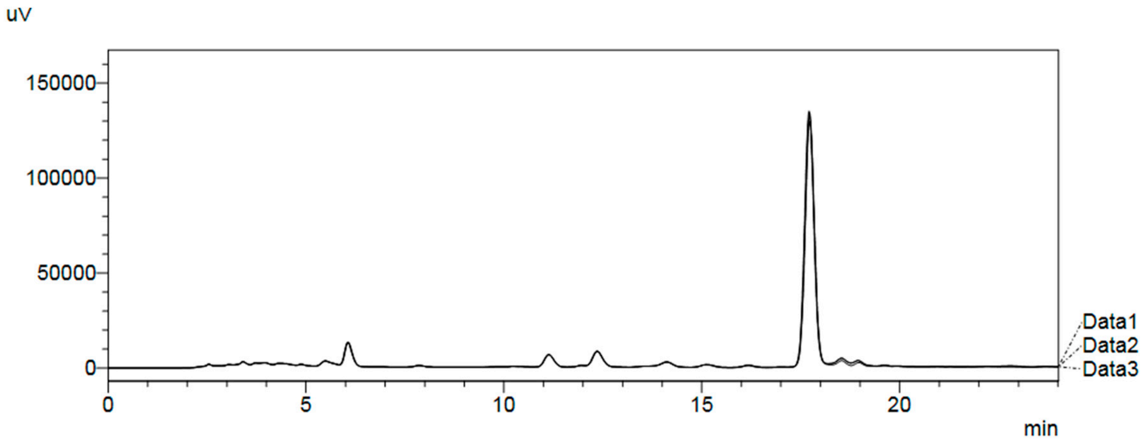

Figure S14. Superimposed chromatograms of rosmarinic acid triplicate (retention times approximately at 17.72 min) for its quantification in November, *Mentha x villosa*.

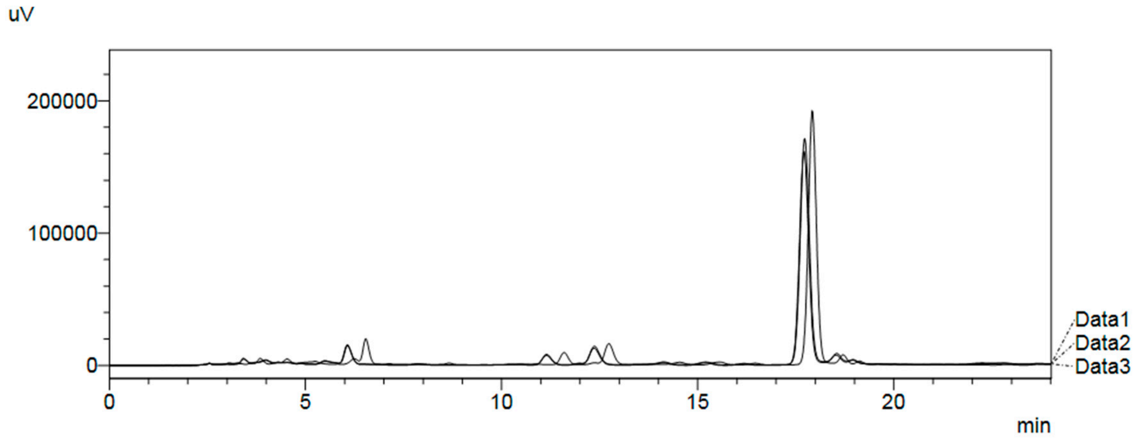

Figure S15. Superimposed chromatograms of rosmarinic acid triplicate (retention times at 17.72; 17.71; 17.92 min) for its quantification in December, *Mentha x villosa*.

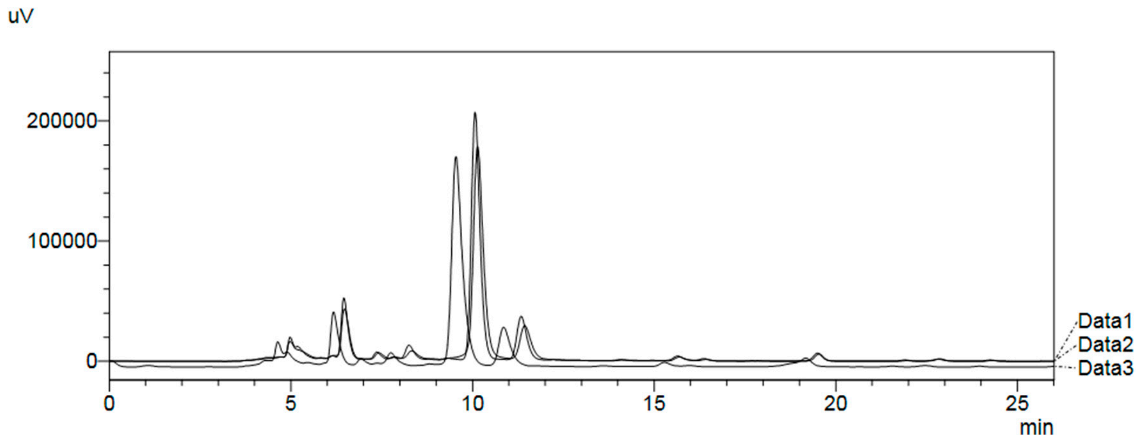

Figure S16. Superimposed chromatograms of rosmarinic acid triplicate (retention times at 10.07; 9.54; 10.07 min) for its quantification in January, *Pectranthus amboinicus*.

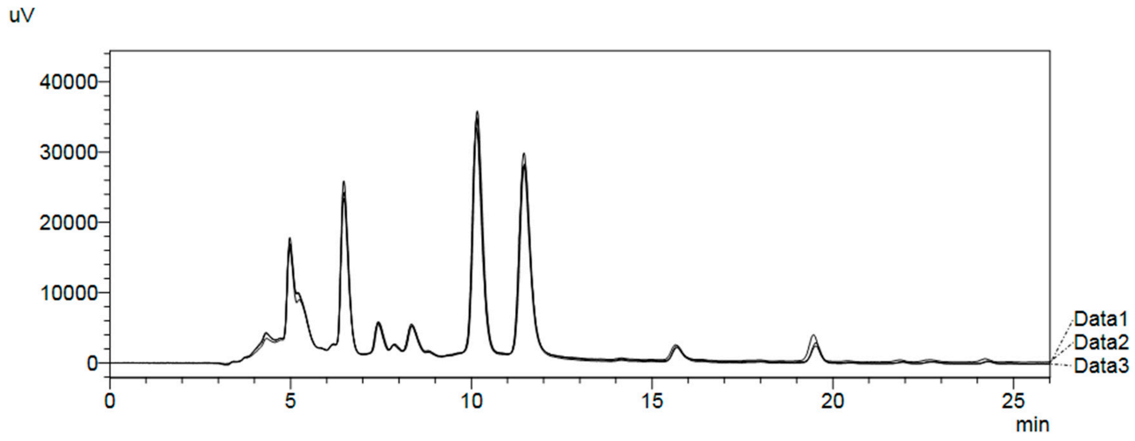

Figure S17. Superimposed chromatograms of rosmarinic acid triplicate (retention times approximately at 10.14 min) for its quantification in February, *Pectranthus amboinicus*.

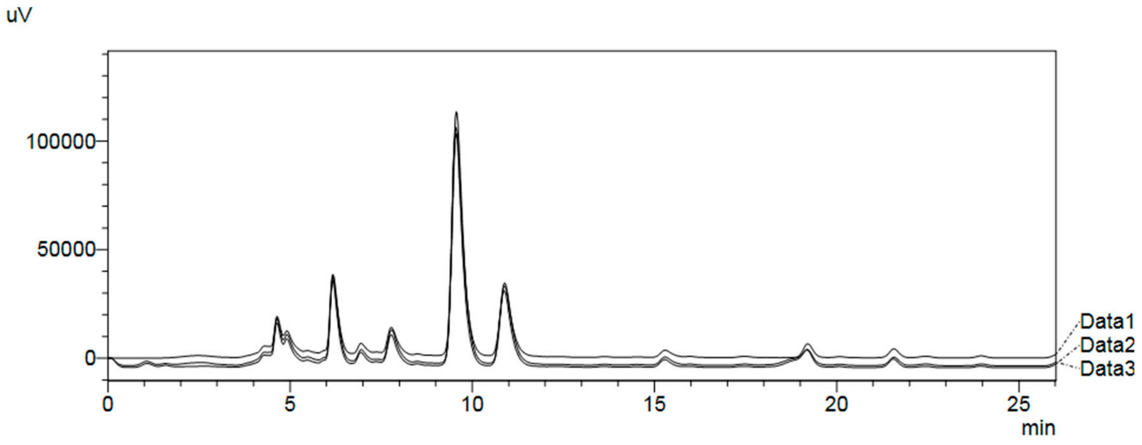

Figure S18. Superimposed chromatograms of rosmarinic acid triplicate (retention times approximately at 9.55 min) for its quantification in March, *Pectranthus amboinicus*.

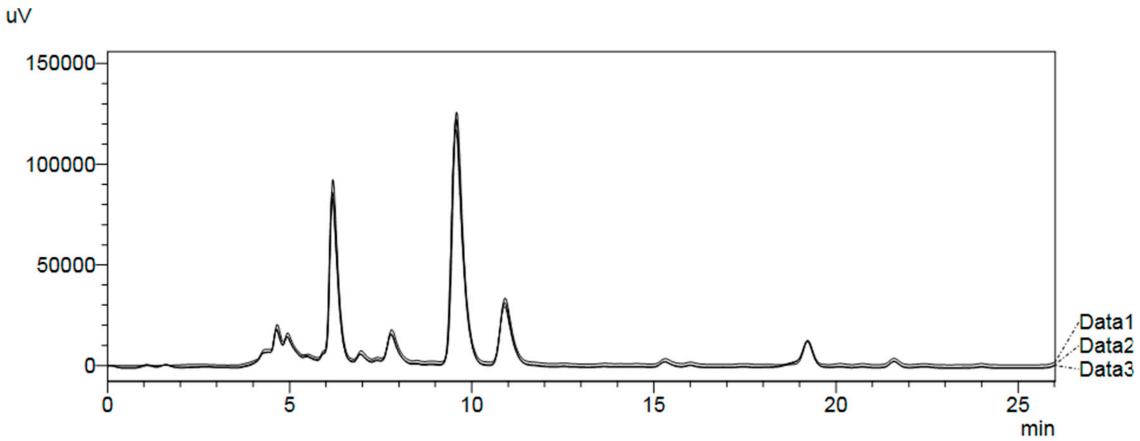

Figure S19. Superimposed chromatograms of rosmarinic acid triplicate (retention times approximately at 9.56 min) for its quantification in April, *Pectranthus amboinicus*.

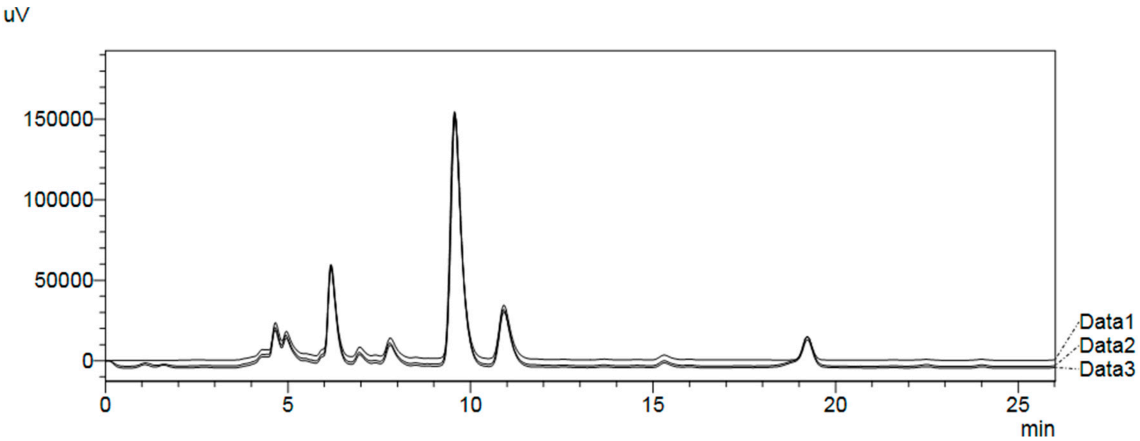

Figure S20. Superimposed chromatograms of rosmarinic acid triplicate (retention times approximately at 9.57 min) for its quantification in June, *Pectranthus amboinicus*.

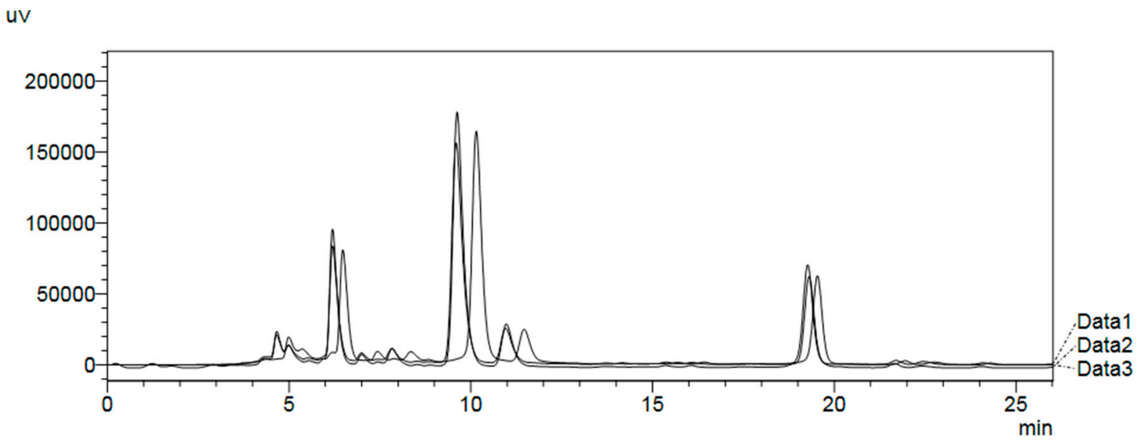

Figure S21. Superimposed chromatograms of rosmarinic acid triplicate (retention times at 9.60; 9.62; 10.15 min) for its quantification in July, *Pectranthus amboinicus*.

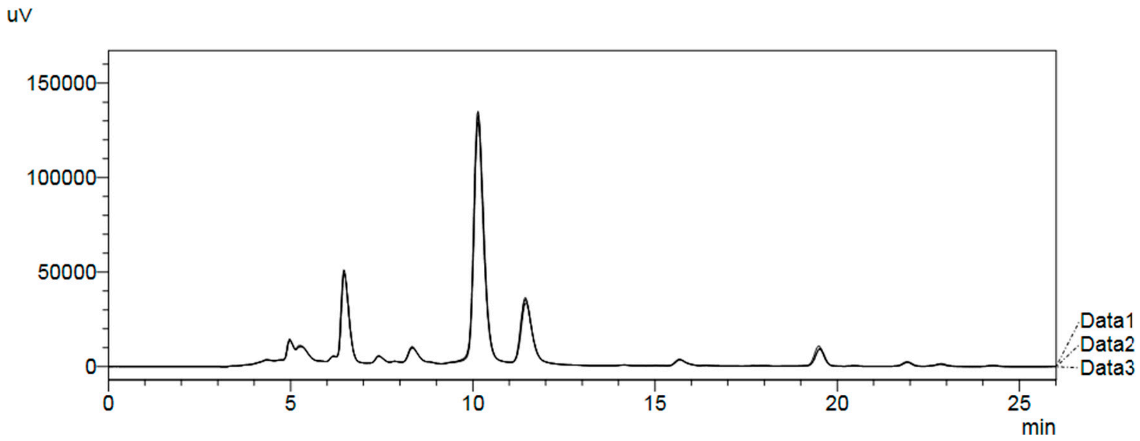

Figure S22. Superimposed chromatograms of rosmarinic acid triplicate (retention times approximately at 10.14 min) for its quantification in August, *Pectranthus amboinicus*.

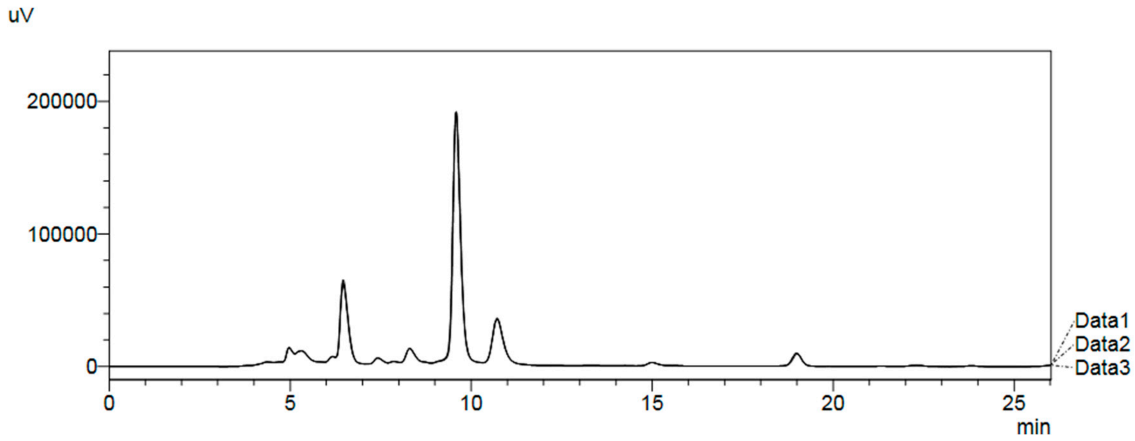

Figure S23. Superimposed chromatograms of rosmarinic acid triplicate (retention times approximately at 9.58 min) for its quantification in September, *Pectranthus amboinicus*.

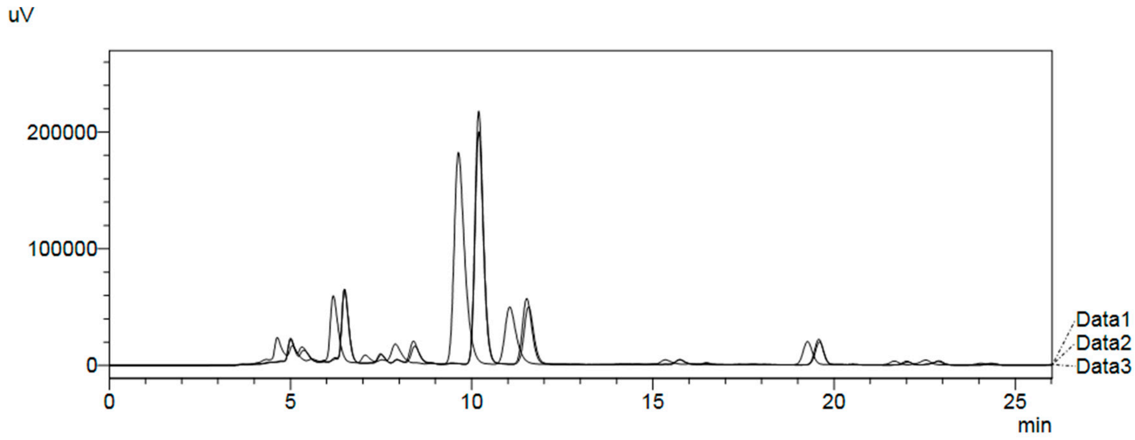

Figure S24. Superimposed chromatograms of rosmarinic acid triplicate (retention times at 9.64; 10.19; 10.19 min) for its quantification in October, *Pectranthus amboinicus*.

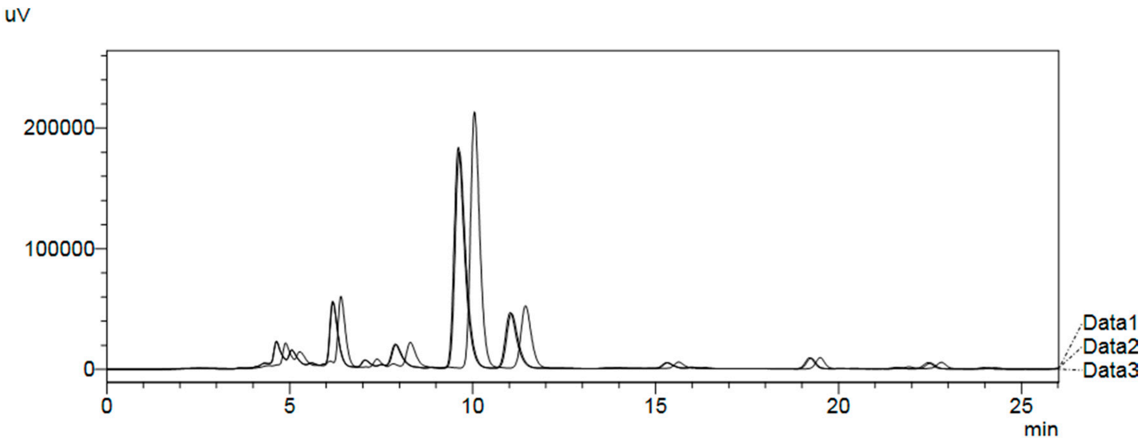

Figure S25. Superimposed chromatograms of rosmarinic acid triplicate (retention times at 10.05; 9.64; 9.61 min) for its quantification in November, *Pectranthus amboinicus*.

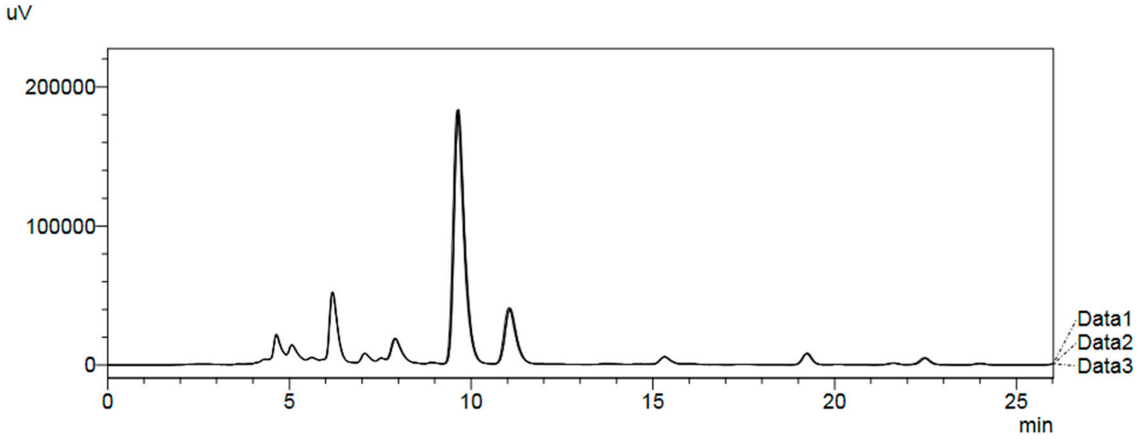

Figure S26. Superimposed chromatograms of rosmarinic acid triplicate (retention times approximately at 9.65 min) for its quantification in December, *Pectranthus amboinicus*.
